# Effects of high rosuvastatin doses on hepatocyte mitochondria of hypercholesterolemic mice

**DOI:** 10.1038/s41598-021-95140-1

**Published:** 2021-08-04

**Authors:** Juan C. Díaz-Zagoya, Alejandro Marín-Medina, Alma M. Zetina-Esquivel, Jorge L. Blé-Castillo, Andrés E. Castell-Rodríguez, Isela E. Juárez-Rojop, Rodrigo Miranda-Zamora

**Affiliations:** 1 División de Investigación, Departamento de Bioquímica, Facultad de Medicina, Universidad Nacional Autónoma de México, CP 04510 Mexico City, Mexico; 2 División Académica de Ciencias de la Salud, Universidad Juárez Autónoma de Tabasco, Villahermosa, Tabasco Mexico; 3 Universidad de Guadalajara, CUCS, Guadalajara, Jalisco Mexico

**Keywords:** Biochemistry, Cardiology, Endocrinology, Medical research

## Abstract

Statins are the cornerstone of therapy for individuals with hyperlipidemia. The aim of this study was to analyze the undesirable effects of mild, moderate and high doses of rosuvastatin in CD-1 male mice who received a cholesterol-rich diet, focusing on the morphological and functional changes on hepatocyte mitochondria. In a mouse model we studied the combined administration of a cholesterol-rich diet along with mild and moderate doses of rosuvastatin (1, 2.5 or 5 mg/kg/day) during several days. After the animals were sacrificed, liver mitochondria were isolated for microscopic studies and to analyze the respiratory function. The respiratory control (state-3/state-4) was evaluated in mice who received high doses of rosuvastatin. Rosuvastatin doses higher than 20 mg/kg/day induced premature death in mice with a hypercholesterolemic diet, but not in mice with a cholesterol-free diet. Doses from 2.5 to 5 mg/kg/day also induced morphological and functional alterations in mitochondria but these hypercholesterolemic animals survived longer. Giving 1 mg/kg/day, which is close to the maximal therapeutic dose for humans, did not affect mitochondrial architecture or respiratory function after two months of treatment. We analyzed the effect of rosuvastatin on hepatic tissue because it is where statins are mainly accumulated and it is the main site of endogenous cholesterol synthesis. Our results contribute to understand the side effects of rosuvastatin in hypercholesterolemic mice, effects that could also affect humans who are intolerant to statins.

## Introduction

Statins are the drugs of choice as first and second prevention of atherosclerosis cardiovascular diseases (ASCVD). The introduction of statins to clinical medicine in 1987^1^ was followed by an extensive therapeutic use for individuals with high serum cholesterol levels in order to control β-containing lipoproteins recognized as ASCVD risk factors^2,3^. Particularly, rosuvastatin is highly used in the clinical practice; 40 mg/day is the highest dose for humans, which means that for an individual weighting 60 kg, the dose is 0.66 mg/kg/day.

Some medical societies have recommended an aggressive use of statins to treat individuals with high ASCVD risk^4–7^. All statins available in the market have the same effect on cholesterol biosynthesis, inhibiting in a competitive way the 3-hydroxy-3-methylglutaryl coenzyme A reductase^8,9^ that catalyzes the formation of mevalonate. This action predominantly affects liver cholesterol synthesis, which is responsible for 80% of its endogenous production in humans^10^. Statins also reduce oxidative stress by modulating the redox systems. This is probably a mechanism by which they exert beneficial effects on the cardiovascular system; however, the oxidative stress may also be responsible for statin-induced adverse effects, such as the actions on glucose homeostasis or producing nephrotoxicity^11^.

As any pharmaceutical compound, statins are not exempted of undesirable side effects, especially after high therapeutic doses during long periods of time, as well as when they are used in individuals with statins intolerance, with a prevalence of 7–29%^12,13^.

There are two types of statins, the natural compounds that were isolated from fungi cultures and the synthetic compounds produced in the laboratory. Synthetic statins are more active and potentially produce more undesirable effects^14^. The solubility of statins also influences their potential accumulation in tissues, as it happens with lipid-soluble compounds^15^.

Animal models can be used to evaluate and compare the side effects of statins^16^. One of the first adverse effects reported is that of lovastatin in rabbits^17^, which was aggravated when a cholesterol-rich diet was added. Some studies have used rodent models to analyze the statins side effects in hypercholesterolemic animals^18,19^. Our study provides data of mice who received a cholesterol-rich diet along with mild, moderate and high doses of rosuvastatin. We particularly looked for adverse effects on liver mitochondria of mice who received a cholesterol-rich-diet in comparison with mice who received a cholesterol-free diet and the same doses of rosuvastatin.

## Material and methods

According to their solubility, the statins employed in patients are divided in two groups, the lipophilic constituted by fluvastatin, lovastatin, simvastatin and pitavastatin, and the hydrophilic integrated by rosuvastatin and pravastatin. It was chosen for this study rosuvastatin that is widely used by clinicians^20^. CD-1 male mice of 30 g body weight were maintained in light–dark cycles of 12 h. They received a laboratory chow diet (CD) containing 18% protein, 5% fat and 5% fiber. The food was powdered and rosuvastatin was added; then, food and statin were administered orally. The hypercholesterolemic diet (HD) contained 2% cholesterol and 0.6% sodium deoxycholate. Rosuvastatin tablets of 20 mg were obtained from Medimart, Slovenia. All other compounds were purchased from Merck-Sigma Mexico.

It was also included CD and HD animals that did not receive rosuvastatin (n = 6 per group). The experimental procedures followed the Official Mexican Standard NOM-062-ZOO-1999 and the guidelines established by the Research Committee for the Care and the Use of Laboratory Animals of the “Universidad Nacional Autónoma de México”.

### Experimental design

A. Mice who received a continuous treatment with rosuvastatin (Ro) for 30 days in order to evaluate their survival rate:

- CD + Ro (rosuvastatin 0, 20, 50, 100, 200, 400 mg/kg/day).
- HD + Ro (rosuvastatin 0, 20, 50, 100, 200, 400 mg/kg/day).

B. Mice who received a continuous treatment with moderate-high statin doses to evaluate hepatocyte mitochondrial respiration. The microscopic observations were performed in representative samples of each group after different treatment lengths for 5 days:

- CD + Ro (rosuvastatin 0, 20 mg/kg/day).
- HD + Ro (rosuvastatin 0, 20 mg/kg/day).

C. Mice who received a continuous treatment with mild doses of statin to evaluate hepatocytes mitochondrial respiration and to perform microscopic observations in representative samples of each group after different treatment lengths for 60 days:

- HD + Ro (rosuvastatin 0, 5 mg/kg/day).
- HD + Ro (rosuvastatin 0, 2.5 mg/kg/day).
- HD + Ro (rosuvastatin 0, 1 mg/kg/day).

### Animal sacrifice

After the treatment indicated in the experimental design, all animals were sacrificed employing a guillotine^21^. Blood and other tissues were immediately obtained for biochemical analysis or microscopic studies.

### Mitochondria isolation and incubation

The liver was homogenized in 250 mM sucrose, 0.5 mM HEPES, 0.5 mM EGTA (SHE) at pH 7.2 and using a Thomas pestle tissue grinder (piston-type Teflon pestle). The liver was homogenized with a soft manual procedure in order to generate the minor damage to mitochondria; then, mitochondria were isolated using a refrigerated centrifuge MPW-353R Med Instruments, Warsaw, following the method described by Frezza et al.^22^. The mitochondria obtained by centrifugation were incubated for 10 min with 0.5% albumin to eliminate fatty acids; then, they were re-suspended in SHE solution. The protein content in mitochondria was evaluated by the Bradford method^23^. The incubation media contained: KCl 240 mM, HEPES 60 mM, H_3_PO_4_ 4 mM, EGTA 4 mM, Succinate 10 mM, MgCl_2_ 4 mM, mitochondrial protein 4 mg, at pH 7.2, final volume 3.2 ml. An YSI oxygen meter 5300 model was used to measure the oxygen consumption. The mitochondrial respiration was stimulated by adding 10 µL of 200 mM ADP^24^. And the respiratory control was evaluated.

### Biochemical parameters

Serum determinations included total cholesterol, triacylglycerols, HDL-C (high density lipoprotein cholesterol), glucose, urea, creatinine, AST (aspartate aminotransferase) and ALT (alanine aminotransferase). We used a semi-automatic equipment of clinical chemistry from Random Access Diagnostics. Besides, the 3-hydroxy-methylglutaryl-CoA/mevalonate ratio was evaluated, this index determines the activity of the enzyme 3-hydroxy-3-methylglutaryl-coenzyme A reductase^25^.

### Microscopy studies

Livers were studied both by light and electronic microscopy. The tissue slices were dyed with hematoxylin–eosin and were observed under a Nikol Eclipse 180 microscope. For electronic microscopy the mitochondrial pellet and liver tissue were fixed with 3% glutaraldehyde in cacodylate buffer. The samples were processed at the microscopy unit of the “Instituto de Neurobiología, Universidad Nacional Autónoma de México”, using a Jeol transmission electron microscope model JEM-1010.

### Statistical analysis

The mice survival was expressed in percentages. One-way analysis of variance (ANOVA), followed by Student–Newman–Keuls test and differences were considered statistically significant when *p* < 0.05. For morphologic analysis we used representative samples from each of the animal groups.

## Results

### Biochemical parameters

**Figure 1:**
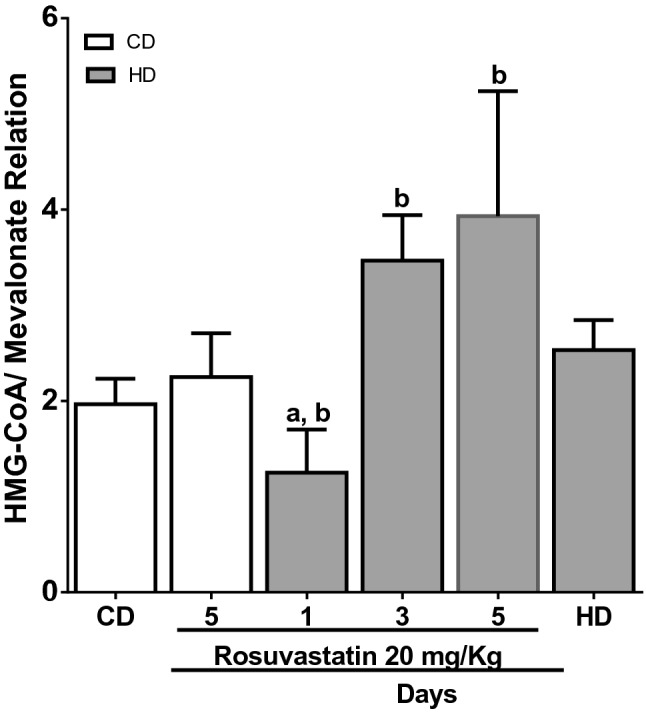
Absorbance of 3-hydroxymethylglutaryl-CoA/mevalonate ratio in the liver homogenate. CD control diet, HD hypercholesterolemic diet. Key of significance: ^a^compared with CD and ^b^with HD (*p* < 0.05).

Serum triacylglycerols decreased after receiving HD + Ro 20 mg/kg/day on day 3 (47 ± 2.82 mg/dL) and day 5 (53 ± 0.80 mg/dL) versus HD without Ro (97 ± 2.82 mg/dL). Conversely, serum cholesterol levels increased significantly on days 3 and 5 (761.5 ± 0.7 mg/dL and 813 ± 1.06 mg/dL, respectively) versus HD without Ro (202 ± 5.65 mg/dL). HDL-C decreased after receiving HD + Ro 20 mg/kg/day on days 3 and 5 (16 ± 1.06 mg/dL and 14 ± 0.14 mg/dL, respectively) compared with HD without Ro (59.2 ± 0.63 mg/dL) (Table 1). An unexpected result was the disturbing decrease in ALT and AST serum activity that was almost undetected on days 3 and 5 after treatment (1.67 ± 0.29 IU/L; 1.93 ± 0.52 IU/L, and 2.33 ± 0.64 IU/L, 2.17 ± 0.60 IU/L; respectively) (Table 1). Regarding the respiratory function, it decreased after 3 and 5 days of receiving HD + Ro 2.90 ± 0.56 and 0.95 ± 0.63) in comparison with HD without Ro (3.05 ± 0.07) (Table 2). The 3-hydroxy-methylglutaryl-CoA/mevalonate ratio, which was high after 3 days of HD + Ro treatment, and even higher after 5 days of treatment (3.93 ± 1.30), versus the HD without Ro group (2.53 ± 0.31) (*p* < 0.05) (Fig. 1). These data reveal that rosuvastatin decreased the activity of 3-hydroxy-3-methylglutaryl-CoA reductase.

**Table 1:** Effect of Rosuvastatin (20 mg/kg/day) in cholesterol, triacylglycerols, HDL-C, and transaminases in serum of mice.

Biochemical parameter	CD	CD + Ro1	HD	HD + Ro1	HD + Ro3	HD + Ro5
Triacylglycerols (mg/dL)	103 ± 1.41	180.5 ± 0.70^a^	97 ± 2.82	131 ± 0.71^b^	47 ± 2.82^b^	53 ± 0.80^b^
Cholesterol (mg/dL)	116.0 ± 1.41	116.0 ± 2.83	202 ± 5.65^a^	165.5 ± 2.12	761.5 ± 0.70^b^	813 ± 1.06^b^
HDL-C (mg/dL)	58.80 ± 1.27	58.35 ± 0.64	59.2 ± 0.63	57.35 ± 1..20	16 ± 1.06^b^	14 ± 0.14^b^
ALT (IU/L)	102.4 ± 5.44	95.58 ± 3.82	113.4 ± 2.24	95.58 ± 3.82	1.67 ± 0.29^b^	1.93 ± 0.52^b^
AsT (IU/L	261.8 ± 1.44	265.8 ± 2.54	275.1 ± 4.34	263.7 ± 1.82	2.33 ± 0.64^b^	2.17 ± 0.60^b^

Triacylglycerols, cholesterol, HDH-C serum level, aspartate aminotransferase (AST), and alanine aminotransferase (ALT), Ro (Rosuvastatin). Values represent the mean ± SEM of eight animals. Statistical analysis was done by One ANOVA, followed by the Student-Newman–Keuls test. Key of significance: ^a^compared with CD and ^b^with HD (*p* < 0.05).

**Table 2:** Respiratory control in hepatocyte mitochondria of mice with cholesterol-rich diet treated with rosuvastatin 20 mg/kg/day.

Group	State 4	State 3	Respiratory control
CD	0.15 ± 0.02	0.53 ± 0.08	3.45 ± 0.07
HD	0.23 ± 0.02	0.72 ± 0.10	3.05 ± 0.07
CD + Ro/5 day	0.19 ± 0.00	0.62 ± 0.07	3.10 ± 0.28
HD + Ro/1 day	0.25 ± 0.03	0.71 ± 0.01	2.80 ± 0.28
HD + Ro/3 days	0.10 ± 0.01	0.30 ± 0.10	2.90 ± 0.56
HD + Ro/5 days	0.09 ± 0.13	0.09 ± 0.13^a^	0.95 ± 0.63^a^

*CD* control diet; *HD* hypercholesterolemic diet; *Ro* rosuvastatin 20 mg/kg/day; Key of significance: ^a^compared with HD (*p* < 0.05).

### Effect of high doses of rosuvastatin on the survival of mice with a cholesterol-rich diet

Table 3 shows the mice survival rate when using different doses of rosuvastatin. The combined administration of Ro and a cholesterol-rich diet produced a premature death with doses 20 mg/kg/day or higher of rosuvastatin. However, there was no-mortality in animals without a cholesterol-rich diet that received the same statin doses. In this context, studies performed by Diaz-Zagoya et al.,^18,19^ showed that high doses of statins administered in a period of 30–60 days in a mouse model without a high-cholesterol diet had a survival rate of 100%.

**Table 3:** Mice survival after high doses of rosuvastatin plus a cholesterol-rich diet, after 30 Days of Treatment.

Ro mg/day	Day							
	0	4	5	6	7	17	18	30
0	100	100	100	100	100	100	100	100
20	100	100	100	100	83	83	66.4	50
50	100	100	50	16.6	16.6	16.6	16.6	16.6
100	100	100	66.4	16.6	16.6	16.6	16.6	16.6
200	100	100	0	0	0	0	0	0
400	100	100	16.6	0	0	0	0	0

*Ro* rosuvastatin, mg/kg/day.

### Effect of moderate doses of rosuvastatin on mice with a cholesterol-rich diet

A 20 mg/kg/day dose of Ro was tested in mice receiving CD or HD. Animals in the HD + Ro group were sacrificed on days 1, 3 and 5 of treatment. A daily analysis was performed in order to observe any morphologic changes in hepatocytes and the respiratory function of mitochondria. The body weigh was registered, as well as the liver weigh in relation to the body weight. Macroscopically, livers of the animal group treated with statins were friable with appearance of steatosis. Table 4 shows that the liver weight of animals who received HD + Ro 20 mg/kg/day increased significantly after 3 and 5 days of treatment. In this regard, previous studies performed by Diaz-Zagoya et al.,^18,19^ showed that no significant changes were observed on day 1 and 3. However, an increase in the weight of the mice liver in relation to body weight was observed in CD + Ro 5 mg/kg/day, this difference was not significant with respect to CD. An increase in the weight of mice liver in relation to body weight was observed in the CD + Ro group, but it was not significant with respect to the CD and HD group.

**Table 4:** Liver weight and its relation to body weight in hypercholesterolemic mice treated with 20 and 2.5 mg/kg/day of rosuvastatin, after days of treatment.

Length of treatment (day)	Liver weight (g; m ± SD)	% of liver weight relative to body weight
**20 mg/kg/day Ro**		
CD	1.71 ± 0.26	9.11
HD	1.85 ± 0.21	5.89
CD + Ro 1	1.86 ± 0.15	7.60
CD + Ro 5	2.28 ± 0.15	8.28
HD + Ro 1	1.65 ± 0.16	7.54
HD + Ro 3 d	2.59 ± 0.79^a^	11.20
HD + Ro 5	3.30 ± 0.62^a^	14.10
**2.5 mg/kg/day Ro**		
HD + Ro 1	1.20 ± 0.27	5.0
HD + Ro 3	1.41 ± 0.39	6.52
HD + Ro 5	1.50 ± 0.31	7–06
HD + Ro 7	2.04 ± 0.38	9.06
HD + Ro 9	2.73 ± 0.71^a^	10.95
HD + Ro 11	2.63 ± 0.79^a^	11.25
HD + Ro 13	2.48 ± 0.25	10.59
HD + Ro 15	2.40 ± 0.35	11.23

*CD* control diet; *HD* hypercholesterolemic diet; *Ro* rosuvastatin 20 mg/kg/day; Ro rosuvastatin 2.5 mg/kg/day, *M* media, *SD* standard deviation. ^a^Key of significance compared with HD (*p* < 0.05).

### Microscopy observations

**Figure 2:**
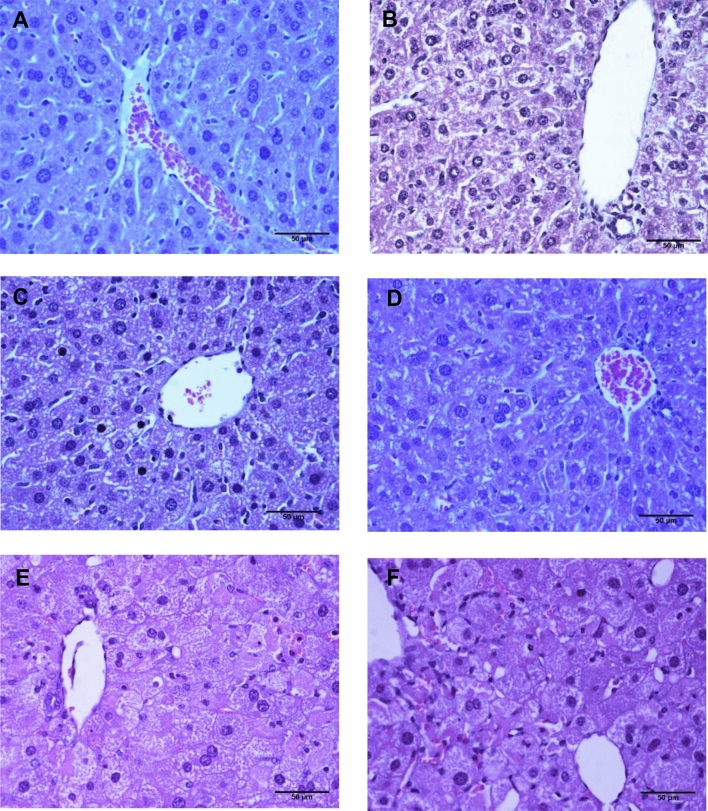
Microscopy images of liver tissue from mice after cholesterol-rich diet and rosuvastatin. Mice liver slices were stained with hematoxylin–eosin. (**A**) CD; (**B**) CD + Ro; (**C**) HD; (**D**) HD + Ro one day; (**E**) HD + Ro 3 days; (**F**) HD + Ro 5 days. CD, control diet; HD, cholesterol-rich diet; Ro, rosuvastatin 20 mg/kg/day. The liver of CD + Ro 20 mg/kg/day obtained after 1, 3 and 5 days of treatment manifest early steatosis and significant changes in the cellular structure. Sinusoidal dilatation (**D–F**), some cells with ballooning degeneration, pyknotic nuclei (**E**,**F**), Mallory Denk bodies (**E**) and hepatocytes with loss of their nuclei (**E**,**F**) are observed.

Liver tissue was stained with hematoxylin/eosin and evaluated after 1, 3 and 5 days of treatment with HD + Ro 20 mg/kg/day. Liver tissue of CD + Ro 20 mg/kg/day (Fig. 2B) or HD (Fig. 2C) showed no alteration compared to liver tissue of CD without Ro (Fig. 2A). Livers of mice who received HD + Ro 20 mg/kg/day, after 1 to 5 days of treatment, showed early steatosis with abundant lipid drops and severe changes such as ballooning degeneration, Mallory Denk bodies and hepatocytes with loss of nuclei. No necrosis was observed (Fig. 2D–F).

### Effect of mild doses of rosuvastatin along with a cholesterol-rich diet

Rosuvastatin doses of 0, 1, 2.5 and 5 mg/kg/day were used during different periods of time. Mice were sacrificed every day and the morphological and physiological evaluations were performed. Animals that received HD + Ro 5 mg/kg/day did not show important differences in their body weight in comparison with other groups; however, their liver weight increased significantly from day 5 on, a fact that was highly apparent when we compared the percentage of liver weight relative to body weight (Table 4). A similar result was obtained with Ro 2.5 mg/kg/day, after 15 days of treatment (Table 4), but not when Ro 1.0 mg/kg/day was administered for 59 days. Treatments with HD + Ro 2.5 and 5 mg/kg/day produced an impaired respiratory function, lower respiratory control and higher oxygen consumption. On day 59 of treatment, a decrease in respiratory control was observed in the Cd + Ro group 1 mg/kg/day (2.96) compared to day 0 in the CD group (3.44) and in the HD + Ro group at 1 mg/kg/day (3.56) on day 59. On the other hand, animals treated with HD + Ro 1.0 mg/kg/day, did not show alterations of the mitochondrial function even after 59 days of treatment (Table 5). It was observed that the HD + Ro 2.5 mg/kg/day group decreased respiratory control (1.80) with respect to the HD group (3.22), this result is similar to the group that received a dose of 5 mg/kg/day (1.79); at the same period of treatment on 5 days (Table 5).

**Table 5:** Oxygen consumption by liver mitochondria in hypercholesterolemic mice treated with rosuvastatin 1.0, 2.5 or 5 mg/kg/day, after 60 Days of Treatment.

Rosuvastatin treatment mg/kg/day	Length of treatment (day)	Respiratory control
CD	0	3.44
HD + Ro 5	3	2.20
HD + Ro 5	5	1.79
HD + Ro 2.5	5	1.80
HD + Ro 2.5	9	2.20
HD + Ro 2.5	15	2.40
HD + Ro 1	59	3.56
HD	59	3.22
CD + Ro 1	59	2.96

*CD* control diet, *HD* hypercholesterolemic diet, *Ro* rosuvastatin.

**Figure 3:**
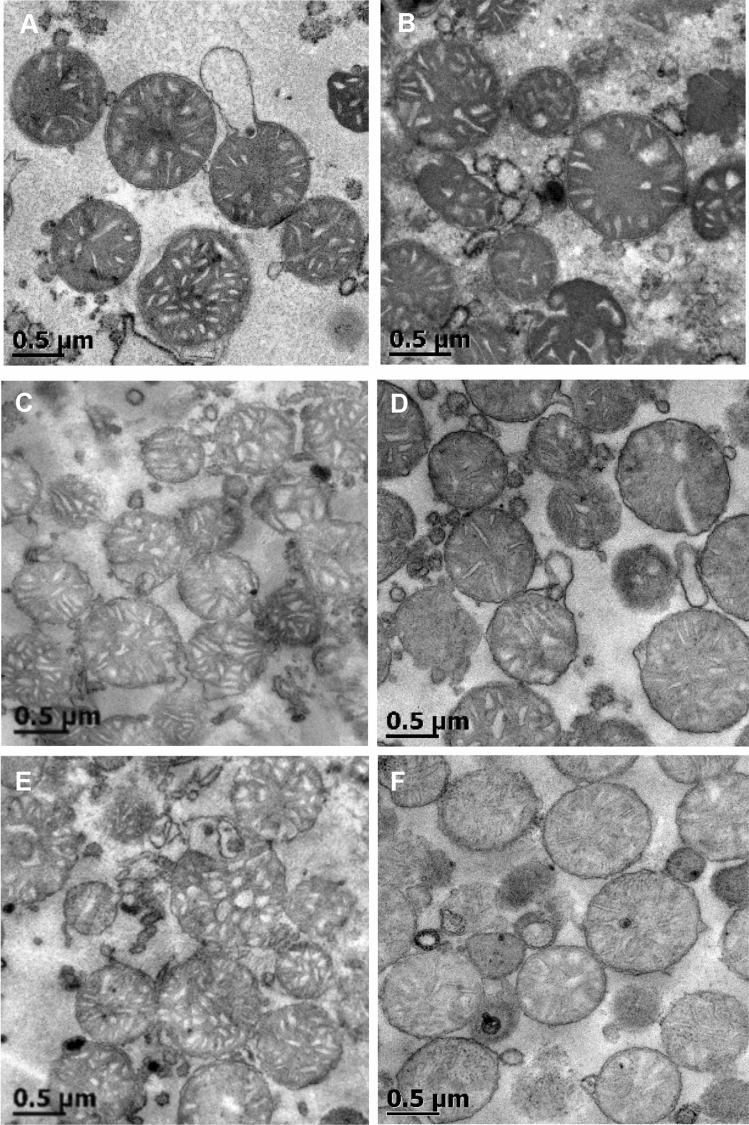
Electron microscopy of hepatocytes mitochondria from mice after cholesterol-rich diet and rosuvastatin. Liver mitochondria from male mice that were treated with (**A**) HD + Ro 5 mg/kg/day 11 days; (**B**) HD + Ro 2.5 mg/kg/day 5 days; (**C**) HD + Ro 1.0 mg/kg/day 59 days; (**D**) CD 59 days; (**E**) CD + Ro 1 mg/kg/day 59 days; (**F**) HD 59 days. CD, control diet; HD, cholesterol-rich diet; Ro, rosuvastatin.

The HD + Ro 5 mg/kg/day treatment (Fig. 3A) was observed to be harmful to mitochondria, showing loss of architecture pattern, some had a donut-like shape and matrixes with empty spaces. The HD + Ro 2.5 mg/kg/day treatment (Fig. 3B) displayed copious mitochondrial damage and disorganized organelle structure. Mitochondria obtained after 59 days of HD + Ro 1 mg/kg/day treatment (Fig. 3C) showed a general conserved structure with few organelles having a horseshoe aspect. Mitochondria in Fig. 3D–F are the corresponding controls.

## Discussion

Rosuvastatin is a powerful synthetic statin that is not exempted of undesirable effects in doses regularly used in humans. If a human of 70 kg body weight, receives a rosuvastatin dose of 40 mg/day, he or she is receiving 0.57 mg/kg/day. We administered different doses to mice, the smallest was equivalent to 1 mg/kg/day; higher doses produced several undesirable effects in our mice model, especially in the liver, the main site of statin accumulation. The 400 mg dose had a 100% mortality after 6 days of treatment, a result that could be explained by the damage, non-compatible with life, produced on mitochondria structure and function; therefore, a moderate-high dose was considered to be 20 mg/kg/day and the mild to moderate doses between 1 and 5 mg/kg/day.

Although some studies have shown that rosuvastatin significantly reduces serum cholesterol concentrations by using lyophilized tablets^26^, we observed an increase in serum cholesterol on days 3 and 5 (761.5 ± 0.7 mg/dL and 813 ± 1.06 mg/dL, respectively) in the HD + Ro group versus the HD without Ro group (202 ± 5.65 mg/dL). Previous studies have already reported that undesirable effects of statins are aggravated if a diet rich in cholesterol is added^17^. The combination of HD + Ro can modify the dynamics of the cholesterol metabolism genes, since when cholesterol concentrations increase, the catalytic release of SREBP2 proteins is blocked, which are important transcription factors for the expression of many genes such as the hepatic LDL receptor (LDLR)^27^. Therefore, having a HD diet could influence the expression of these genes by decreasing the amount of LDLR in the liver, so the amount of cholesterol that reaches the liver could be reduced and redistributed in plasma and other tissues.

Other studies have reported an increase in serum cholesterol due to the use of rosuvastatin, it has been observed that mice treated with Ro had an increase in total serum cholesterol; and although Ro has been observed to cause an increase in hepatic LDL receptor messenger RNA in humans, the opposite effect has been observed in rats^28^. Therefore, the amount of this protein in the liver decreases and the serum concentration of cholesterol increases. This result supports the increase in serum cholesterol found in our study from the third day of treatment with Ro; therefore, this double effect could explain the increase in cholesterol in mice treated with HD + Ro.

Undesirable side effects have also been reported in human skeletal muscle where myositis and even rhabdomyolysis have been observed^29^. Although the use of low-dose statins helps to restore the histological architecture of the liver in individuals with NAFLD^30^, it has also been observed that in individuals with complicated sepsis, statins appear to accelerate liver damage. In this sense, we observed that Ro at low doses (1 mg) did not significantly affect respiratory function, even in a prolonged treatment (59 days) compared with doses of 5 and 2.5 mg that significantly affected respiratory function from day 5. A study on mice with dystrophin protein deficiency who received 10 mg/kg of rosuvastatin, showed significantly increased areas of myonecrosis and inflammation, while mice without altered dystrophin who received the same doses showed increased levels of NF-Κβ, TNF-α as well as increased creatine kinase activity^31^, which suggests that high doses of rosuvastatin could potentiate the expression of some genes that encode proteins of inflammation pathways. In this sense, some authors presented that Rosuvastatin increases the expression of IL-10, and inhibits the overexpression of NF-κB^32,33^. Besides, there is evidence to suggest that statins inhibit the Myd88 pathway and therefore suppress the NF-κB pathway^34^.

Besides the cholesterol-lowering effect of statins, it has been suggested that statins can be used as an associated therapy for COVID-19 due to their anti-inflammatory action that might reduce the risk of cardiovascular complications caused by SARS-CoV-2 virus^35,36^. Therefore, rosuvastatin and other statins are under a close examination.

In this study, we observed that high doses of rosuvastatin affected liver mitochondria architecture and mitochondrial respiration. We compared the oxygen consumption in states 3 and 4 of respiration; phosphorylation in state 3 was stimulated by adding 200 mM ADP to the incubation medium. The state 3/state 4 ratio decreased when mice received rosuvastatin doses of 2.5 mg/kg/day or higher. Previous studies using high doses of simvastatin in rats and humans have reported a decreased function of complex I in the respiratory chain^37^ which considerably decreases the flow of electrons from NADH towards complex III. Therefore, under these conditions the mitochondrial energy load would depend mostly of the electron flow from FADH to complex II, resulting in a lower production of ATP by the ATP synthase. Additionally, the inhibition of the 3-hydroxy-3 methyl glutaryl CoA reductase decreases the formation of coenzyme Q^38^, essential for the transport of electrons to complex III and would affect the Q cycle in this complex, which is important for the regeneration of coenzyme Q. All these factors together contribute to a decrease in ATP synthesis.

Animal model studies have explored diseases that affect mitochondrial function altering the transport of metabolites to mitochondria (especially ADP)^39^. We did not explore the intimal mechanism of the respiratory chain alteration, but there is information regarding the changes that other statins induce in muscle mitochondrial function^40–42^. Moreover, a decrease in mitochondrial respiration was observed in rats treated with low doses of Rosuvastatin 1 mg/kg/day (not statistically significant); and a small decrease in respiration of the mitochondrial complex was also reported at the same doses, but without affecting the functioning of this complex^37^. We observed that the 3-hydroxy-3-methylglutaryl-CoA/mevalonate ratio in liver homogenate was high on day 3 and higher on day 5 of treatment; this result shows the inhibitory action of Ro on the HMG-CoA reductase enzyme. This inhibitory action of Ro on the main regulatory site of cholesterogenesis was not able to overthrow the effect of the cholesterol-rich diet on serum cholesterol levels.

The role of several molecules in inflammatory processes at mitochondrial level is already known. For instance, NF‐Κβ plays a very important role in many mitochondria functions and it has been observed that its over-activation is related to inflammatory processes and cancer^43^. Furthermore, NF‐Κβ is closely related to the functioning of antioxidant proteins expressed in mitochondria such as superoxide dismutase, catalase and glutathione peroxidase, which are important enzymes in the regulation of free radical genesis and proper functioning of mitochondrial energy metabolism^44^. On the other hand, rosuvastatin has been reported to have beneficial cardioprotective effects in mice who received mild doses; in high doses however, this effect is lost and even harmful effects have been observed^45^.

Rosuvastatin is minimally metabolized through CYP 450 enzymes^11^. An abnormal drug metabolism produces a large number of free radicals and lipid peroxidation resulting in oxidative stress and cells damage. In studies carried out in animal models, it was observed that the administration of statins in high doses for 8 weeks, decreased the activity of several enzymes related to oxidative stress (SOD, GHS and GR); therefore, a decrease in the activity of these enzymes could increase the production of free radicals. Besides, other additional mechanisms that may contribute to statin-induced oxidative stress have been observed: decreased activity of transporters (ABCB1 and ABCG2), activation of the caspase pathway, and decreased synthesis of coenzyme Q[^11,46^]. In this sense, Rosuvastatin reduces serum lipids and cholesterol, resulting in CoQ_10_ deficiency; CoQ_10_ is the antioxidant that can protect biological membranes from free radicals and lipid peroxidation, and CoQ_10_ deficiency might be traumatic after high doses of statins. In our study, a decrease in HDL-cholesterol was observed from day 3 of treatment with doses of 20 mg/kg/day; the dose-dependent effect of statins on LDL cholesterol reduction has previously been reported^47,48^. However, another study showed a low increase in HDL-C and a decrease in the capacity for cholesterol efflux by the ABCA1 protein after treatment with atorvastatin^49^, which is important in the formation of HDL lipoproteins. Therefore, rosuvastatin in high doses could significantly affect the efflux of cholesterol by the ABCA1 protein and thus reduce the amount of HDL-C.

In electron microscopy, we observed rosette-shaped mitochondria, alterations in the inner membrane continuity and matrix with vacuous spaces. In light microscopy, hepatocytes with ballooning degeneration and cells with Mallory-Denk bodies were observed; from day 3 of treatment with HD + rosuvastatin at 20 mg/kg/day, these changes occurred in response to a severe depletion of ATP^50^ and represent the macroscopic modifications that high doses of rosuvastatin have on mitochondrial metabolism.

## Conclusion

HD + Ro in doses of 20 mg or higher produced premature death in our model; high rosuvastatin doses given to hypercholesterolemic mice produced severe changes in mitochondrial architecture and function, while low doses did not produce relevant changes in mitochondrial function or architecture. The combination of HD + Ro in our model increased the concentration of serum cholesterol and decreased that of HDL-C. More studies on mitochondrial alteration by the combination of rosuvastatin in a cholesterol-rich tissue are needed to translate these findings into humans.

### Statement of methods

All methods were performed following the relevant guidelines and regulations established by the General Direction of Academic Matters (DGAPA-IN213718) of the “Universidad Nacional Autónoma de México”.

### Statement and ethical considerations of experimental protocols in animal models

The animal study protocol was approved by the Research Committee for the Care and the Use of Laboratory Animals of the “Universidad Nacional Autónoma de México” (209-64).

### Statement of study

The present study was performed according to the ARRIVE guideline.

## Supplementary Information

Supplementary Information.
