# Supplementary material for: Effects of high rosuvastatin doses on hepatocyte mitochondria of hypercholesterolemic mice
Source: Sci Rep. 2021 Aug 4;11:15809. doi: 10.1038/s41598-021-95140-1 (PMC8338935; doi:10.1038/s41598-021-95140-1)
Supplement: Supplementary file 1 — Supplementary Information. [file 41598_2021_95140_MOESM1_ESM.doc]

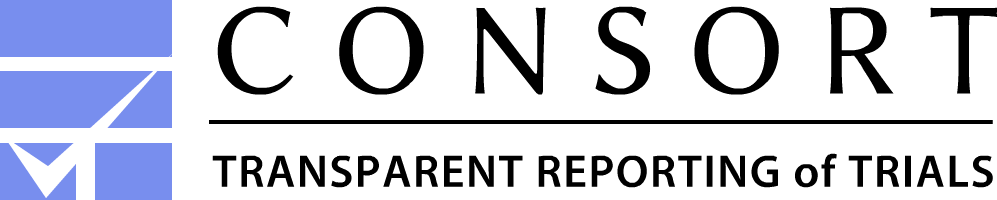
**CONSORT 2010 Flow Diagram.** Evaluation of the effect of mild, moderate and high doses of rosuvastatin (0, 1, 2.5, 5, 20, 40, 100, and 400 mg/kg/day) with respect to the treatment period (5, 30 and 60 days) in mice with a high cholesterol diet

**Allocation**

**Analysis**

**Enrollment**

**Follow-Up**

**30 days**

**60 days**

**5 days**

CD-1 male mice; body weight 30g (n= 130)

Excluded (n=10)

♦Not have the weight (n=10)

Analyzed (n= 60 ) **Treatment A**

♦Excluded from analysis (n= 0 )

Allocated to intervention: **Treatment A** (n=60)

♦Group I: CD+Ro (0, 20, 50, 100, 200, 400 mg/kg/day)

(n=30)

♦Group II: HD+Ro (0, 20, 50, 100, 200, 400 mg/kg/day)

(n=30)

Lost to follow-up (n= 0 ) **Treatment A**

Discontinued intervention (n= 0 )

Allocated to intervention: **Treatment C**

(n=36)

♦Group I: HD+Ro (0, 5 mg/kg/day)

(n=12)

♦Group II: HD+Ro (0, 2.5 mg/kg/day)

(n=12)

♦Group III: HD+Ro (0, 1 mg/kg/day)

(n=12)

Randomized (n=120)

Allocated to intervention: **Treatment B**

(n=24)

♦Group I: CD+Ro (0, 20 mg/kg/day)

(n=12)

♦Group II: HD+Ro (0, 20 mg/kg/day)

(n=12)

Lost to follow-up (n= 0 ) **Treatment B**

Discontinued intervention (n= 0 )

Lost to follow-up (n= 0 ) **Treatment C**

Discontinued intervention (n= 0 )

Analyzed (n= 24) **Treatment B**

♦Excluded from analysis (n= 0 )

Analyzed (n= 36) **Treatment C**

♦Excluded from analysis (n= 0 )

Blood and liver were immediately obtained for:

♦ Biochemical analysis in serum

♦ Microscopic studies: Livers were studied by light and electronic microscopy.

♦ Liver mitochondria: respiratory function
